# The impact of late follicular progesterone level on in vitro fertilization-intracytoplasmic sperm injection outcome: Case-control study

**DOI:** 10.18502/ijrm.v13i5.7157

**Published:** 2020-05-31

**Authors:** Diana Novia, Hilma Putri Lubis, Binarwan Halim, Anantya Pustimbara, Retno Lestari, Abinawanto Abinawanto, Anom Bowolaksono

**Affiliations:** ^1^Department of Biology, Faculty of Mathematics and Science, University of Indonesia, Depok, Indonesia.; ^2^Halim Fertility Center, Stella Maris Women and Children Hospital, Medan, Indonesia.; ^3^Faculty of Medicine University of Sumatera Utara, Haji Adam Malik General Hospital, Medan, Indonesia.

**Keywords:** Intracytoplasmic sperm injection, Embryo, Progesterone, In vitro fertilization.

## Abstract

**Background:**

Studies have been conducted to improve the pregnancy rate through the in vitro fertilization (IVF) and intracytoplasmic sperm injection (ICSI) program. In recent years, researchers have been focusing on finding impact of high progesterone level on endometrial receptivity. However, data on whether progesterone level also affects the quality of the embryo is still limited.

**Objective:**

The aim is to assess the effect of late follicular progesterone level on the outcome of in vitro fertilization-intracytoplasmic sperm injection (IVF-ICSI).

**Materials and Methods:**

This was a case-control of 245 women who underwent in vitro fertilization cycle at Halim Fertility Center, Indonesia. The outcomes assessed were number of oocytes retrieved (OR), maturation rate (MR), fertilization rate (FR), number of good embryos (GE), number of fair embryos (FE), and number of poor embryos (PE). The progesterone (P4) and estradiol (E2) levels were analyzed on the day of human chorionic gonadotropin injection. Serum progesterone level was divided into three groups: 1. low progesterone (≤ 0.50 ng/ml), 2. normal progesterone (0.51-1.50 ng/ml), and 3. high progesterone (> 1.50 ng/ml). All outcomes were compared amongst the groups.

**Results:**

Significant differences occurred between progesterone level on the day of human chorionic gonadotropin administration. The number of OR in group 1, 2, and 3 were 8.41 ± 5.88 vs. 12.99 ± 8.51 vs. 17.58 ± 9.52, respectively.

**Conclusion:**

Progesterone level on the day of human chorionic gonadotropin injection may have an impact on the outcome of IVF-ICSI.

## 1. Introduction

Infertility can be described as a condition where married couples fail to conceive clinically after one year of regular and unprotected sexual intercourse (1, 2). There are about 8-12% or 50-80 million couples around the world who experience infertility (2). Several methods can be used to help infertile couples conceive. The methods used can be in the form of drugs such as clomiphene citrate or procedures such as intrauterine insemination (IUI) and assisted reproductive technology (ART) such as in in vitro fertilization (IVF) or in intracytoplasmic sperm injection (ICSI) (3). About 10% of the infertile couples need the help of an ART to conceive. It is known that as much as 40% of infertility is caused by female factors, 40% by male factors, and the remaining 20% is caused by both or any unexplained factors (4).

According to the practice committee of the American Society for Reproductive Medicine (5), infertility in female can be caused by a few factors, such as ovulation disorders where there are abnormalities in the female's menstrual cycles, tubal and pelvic disorders that are caused by infection and endometriosis, and uterine disorders that include submucosal myomas, endometrial polyps, leiomyomas, and Asherman's syndrome. Meanwhile, the infertility in male often occurs due to the poor quality of sperm.

Since the birth of Louise Brown in 1978, many researches have been conducted to improve the pregnancy rate through IVF program. In recent years, researchers have been focusing on finding the relationship between serum progesterone level on the day of human chorionic gonadotropin (hCG) administration and endometrial receptivity or live birth rate (6-9). Elevated serum progesterone level on the day of hCG injection is known as premature luteinization (PL) and is frequently found in GnRH antagonist cycle. PL on the day of hCG injection has a negative effect on clinical pregnancy rate probably due to embryo-endometrial asynchrony (10).

The assessment of oocyte and embryo quality based on the progesterone serum levels on the day of hCG injection has been rarely studied with a very limited number of cases (7, 11). Some studies state that there is no association between progesterone elevation level and fertilization rates (FRs) as well as the oocyte and embryo quality (12, 13). However, Huang *et al*. stated that serum progesterone concentration on the day of hCG injection is positively and significantly correlated with oocyte fertilization failure (9).

Since there are still very few studies on the effect of progesterone level on oocyte and embryo quality, hence, this study aims to assess the effect of late follicular progesterone level on the outcomes of IVF-ICSI. The outcomes assessed will be the number of oocytes retrieved (OR), maturation rate (MR), fertilization rate (FR), number of good embryos (GE), number of fair embryos (FE), and number of poor embryos (PE).

## 2. Materials and Methods

### Study design

A case-control study was conducted among infertile couples who underwent IVF-ICSI cycle at the Halim Fertility Center, Stella Maris Women and Children Hospital, Indonesia, from January to August 2018. The inclusion criteria in this study were all infertile couples who underwent IVF-ICSI at the center, and the exclusion criteria was women who did not check progesterone and estradiol serum at the time of hCG injection. A total of 245 infertile women who underwent IVF cycle during the period in which serum P4 level was measured on the day of hCG injection were included in the study. The outcomes assessed were number of OR, MR, FR, GE, FE, and PE. The embryo quality was measured based on Istanbul consensus, ALPHA Scientist in Reproductive Medicine & ESHRE Special Interest Group Embryology (14).

### Ovarian hyperstimulation controlled 

The standard protocol for controlled ovarian stimulation was carried out by administering a recombinant follicle-stimulating hormone (FSH) (Gonal-F; Serono; Switzerland) at a dose of 150-300 IU. This protocol used on day 2 in the menstrual cycle. When at least one follicle had reached >14 mm, a gonadotropin-releasing hormone (GnRH) antagonist, Cetrotide (Serono; Switzerland), was used to prevent a premature luteinizing hormone (LH) surge at 0.25 mg dose. The follicular growth was monitored by transvaginal ultrasound since day 4 of the gonadotropin injection, with the average of stimulation duration was 8-10 days. A recombinant hCG (Ovidrel; Serono; Switzerland) was administered to trigger oocyte maturation when three lead follicles had reached 17-18 mm, at least. Oocytes were then retrieved 35-36 hr after hCG injection using an ultrasound-guided transvaginal needle aspiration of follicles.

### Oocyte morphology assessment and ICSI procedure

Cumulus-oocyte complexes (COC) retrieved were washed in MOPS-buffered medium with human serum albumin (GMOPS Plus; Vitrolife; Sweden). The COC were cultured in 0.8 ml GIVF medium (Vitrolife; Sweden) under paraffin oil at 37°C, 6% CO${}_{2}$ and 5% O${}_{2}$ for 2 hr after retrieval. After 2 hr of incubation, COC were then exposed to hyaluronidase (80 IU Synvitro Hyadase; Medicult; Denmark) in order to remove the cumulus cells. Metaphase II oocytes were inseminated using the ICSI method 38-42 hr after the hCG injection. Prior to ICSI, each oocyte was set into a 15 μl droplet of GMOPS Plus (Vitrolife; Sweden) under paraffin oil (Ovoil; Vitrolife; Sweden). Injected oocytes were cultured in a single droplet of 50 μl GTL (Vitrolife; Sweden) until 3 days.

On the day of oocyte(s) retrieval, spouse collected the sperm samples using masturbation method. The semen samples were analyzed for concentration, motility, as well as morphology. The semen was processed and selected by using a density gradient method, layer concentration used was 45:90% SpermGrad (Vitrolife; Sweden). The solution was centrifuged at 300-600 g for 10-20 min and re-suspended with 1 ml of MOPS-buffered medium (GMOPS Plus, Vitrolife; Sweden). For ICSI purposes, a single spermatozoon was immobilized using a polyvinylpyrrolidone (PVP) solution (Medicult; Denmark).

### Embryo morphology assessment

Embryo fertilization was appraised 17 ± 1 hr after the ICSI with the presence of two pronuclei and second polar bodies. Day 3 embryo quality check was assessed 68 ± 1 hr after the ICSI. Embryo quality was assessed morphologically by considering the number of blastomeres, degree of fragmentation, cell size, and presence of multinucleation. Embryo morphology was then classified as good, fair, and poor. Embryos with six to eight equally sized mononucleotide blastomeres and fragmentation < 10% were scored as good. Those with six to eight unequally sized mononucleotide blastomeres and fragmentation between 10-25% were scored as fair. Embryos with severe fragmentation (> 25%), unequal size of blastomeres, and with multinucleation were scored as poor.

### Hormones measurements

Blood sampling was done in the morning of the day of hCG injection for the measurement of progesterone and estradiol level. The serum progesterone and estradiol were analyzed using a microparticle enzyme immunoassay (Vidas; bioMérieux SA) system. The progesterone level was then divided into three groups: group 1 with low progesterone (≤ 0.50 ng/ml), group 2 with normal progesterone (0.51-1.50 ng/ml), and group 3 with high progesterone (> 1.50 ng/ml). All outcomes were compared amongst groups.

### Ethical consideration

Each couple which enrolled in the study signed a written informed consent. The study was authorized by the Ethics Committee of the Stella Maris Women and Children Hospital (1142-1/Dir/RSIA.SM/1/2018), Indonesia.

### Data analysis

We tabulated data on subjects' age and Body Mass Index (BMI) as demographics of subjects. Oocyte retrieved, maturation rate, and embryo quality were tabulated as treatment protocol and outcomes. Estradiol level was tabulated as endocrine profile. Data were analyzed statistically using a Wilcoxon test. Results were then expressed as mean ± SD. A p-value of < 0.05 was considered to be statistically significant. Data were analyzed using software IBM SPSS Statistics (Version 25) for the data analysis process.

## 3. Results

A total of 245 women underwent in vitro fertilization cycle at the Halim Fertility Center, Stella Maris Women and Children Hospital, Indonesia, from January to August 2018. All subjects were then divided into three groups based on their serum progesterone level analyzed in the morning of the day of hCG injection: group 1 with low progesterone (≤ 0.50 ng/ml), group 2 with normal progesterone (0.51-1.50 ng/ml), and group 3 with high progesterone (> 1.50 ng/ml).

As shown in Table I, the subjects that mainly belonged to group 2 had a higher cycles numbers compared to other groups (n = 117). The age range of subjects were 22-47 yr and the mean age is as shown in Table I. The mean body mass index of the three groups was 25.26 ± 3.88 kg/m${}^{2}$; 25.00 ± 3.72 kg/m${}^{2}$; and 24.40 ± 3.51 kg/m${}^{2}$, respectively.

As shown in Table II, the number of OR was significantly higher in group 3 compared to the other groups. It also shows that oocytes in group 1 had a significantly higher maturation and FRs (75.78%; 76.12%, respectively; p = 0.001) but had the lowest percent of GE (p = 0.009). Group 2 had the highest percent of FE and also the lowest FR (p = 0.001). Although group 3 had the lowest maturation rate compared to the other groups, it had a significantly higher percent of GE. Apart from the low maturation rate and the high percent of GE, group 3 also had the highest percent of PE compared to the other groups although it was not significant (p = 0.205). The estradiol serum level on the day of hCG injection was 1247.09 ± 730.56, 2092.39 ± 1276.42, and 3266.43 ± 319.06 in group 1, 2, and 3, respectively where the highest level is in group 3 (p = 0.001).

**Table 1:** Demographics of the subjects


	**Group 1**	**Group 2**	**Group 3**
**Cycles (n)**	64	117	64
**Age (yr)**	35.27 ± 4.85	33.32 ± 4.60	32.31 ± 4.21
**BMI (kg/m ${}^{2}$)**	25.26 ± 3.88	25.00 ± 3.72	24.40 ± 3.51
*BMI: Body mass index			

**Table 2:** Treatment protocol and outcomes


	**Total**	**Group 1**	**Group 2**	**Group 3**	**P-value**
Oocyte retrieved*	12.99 ± 8.82	8.41 ± 5.88	12.99 ± 8.51	17.58 ± 9.52	0.001
Maturation rate**	72.86	75.78	72.44	70.71	0.001
Fertilization rate**	72.25	76.12	70.69	71.21	0.001
Good embryo**	23.09	21.33	20.36	29.01	0.009
Fair embryo**	51.53	59.11	52.74	43.68	0.001
Poor embryo**	25.37	19.56	26.88	27.30	0.205
*Data presented as Mean ± SD. Wilcoxon test, **Data presented as percentages					

## 4. Discussion

Our result suggests that progesterone level on the day of human chorionic gonadotropin injection may have an impact on the outcome of in vitro fertilization IVF-ICSI. Oocytes are not able to express gonadotropin to induce their own maturation. Hence, LH is responsible in activating theca cells and granulosa cells so that oocytes can return to the meiotic stage and begin the maturation process (15). Human chorionic gonadotropin (hCG) is a homologue of LH which can activate LH receptors, a primary, and also the most commonly used, trigger for oocyte maturation (16). In IVF, hCG is a hormone used to create an expression for LH-like exposure. The influence of serum progesterone level on the day of hCG injection on the outcome of the IVF program has long been a matter of debate.

In this study, we found a significant difference in the serum progesterone level with regards to oocyte maturation and FRs among the groups. This result is in line with a recent study which stated that serum progesterone concentration on the day of hCG injection was positively and significantly correlated with the rescue ICSI rate (9). A high progesterone level (> 1.50 ng/ml) on the day of hCG injection may have negatively affected oocyte fertilization, which indicates that greater attention will be needed to avoid fertilization failure. Some studies have reported that an elevation of serum progesterone level as a result of premature oocyte maturation or aging may reduce FR. This oocyte aging may lead to disorganization of the oocyte spindle and cytoplasm as well as the retention of the second polar body which may result in chromosomal anomalies (9).

**Figure 1:**
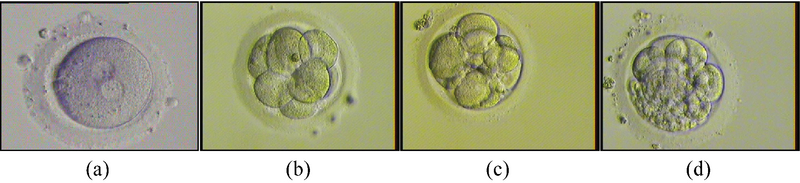
Embryo morphology assessment in Group 3. (a) Fertilization results in the presence of two pronuclei in oocyte and second polar bodies seen in perivitelline space. (b) The good embryo has blastomeres of equal size with no fragmentation. (c) Fair embryo has unequal sized blastomeres and fragmentation between 10 and 25%. (d) The poor embryo has a severe fragmentation and unequal sized blastomere.(×400 magnification).

Our study also showed that high level of progesterone on the day of hCG injection did not have an adverse effect on embryo quality. As shown in Figure 1, embryo in group 3 can also develop into a good-quality embryo. In contrast, however, some study showed that high progesterone level may have detrimental effects on oocyte and embryo quality (17, 18). Sonigo *et al.* (13) stated that embryo cleavage and blastocyst rate are not affected by the elevated progesterone level. Slight impact of the elevated progesterone level on oocyte or embryo quality is found consistent with the evidence that female gametes do not express progesterone receptor. Previous study showed that the quality of embryo is mostly determined by the quality of oocytes, which include the first polar body, meiotic spindle, cumulus cells, and mitochondria (19).

Women who are high responders of gonadotropin, with > 10 follicles in the size of > 14 mm and estradiol level of > 2500 pg/ml on the day of hCG injection may also have a significantly high rise of progesterone. Serum progesterone level on the day of hCG injection reported closely related to the dose of gonadotropin injected irrespectively to the type given. Some study also explained that this event is able to occur due to the initial intense recombinant FSH dose administered during the first six days of stimulation, which in return, may increase the steroidogenetic activity of granulosa cell. According to the study conducted by MERiT, the rise of serum progesterone appears to be more often in the FSH recombinant treatment group compared to the HMG treatment group (10, 20).

PL may negatively impact the success rate of IVF/ICSI fresh transfer, not through an ovarian event but its influence on the endometrium, which may possibly lead to impaired endometrial receptivity. Elevated progesterone level may cause embryo/endometrial asynchrony, which in return may reduce the probability of implantation (21). Hence, clinicians will need to make use of some strategies in controlling PL with the aim of avoiding its possible deleterious effects in fresh IVF cycles. One of the strategies to improve the pregnancy rate in PL cases is to cancel the fresh ET and replacing the fresh embryo with frozen-thawed embryo (22).

The limitation in this study is related to the number of samples. Due to the short duration of the study, we only involved 245 women. Since there are some points in the study that are still being debated, hence, further research with a longer duration is needed to improve the result of this study, which allows the researcher to recruit more samples. For further research, we would like to suggest a study to assess the association between progesterone receptor polymorphism and the outcome of IVF.

## 5. Conclusion

Increased progesterone level on the day of follicle maturation may not affect embryo quality but may have an adverse effect on oocyte maturation and FRs. Elevated estradiol and progesterone level on the day of hCG injection were the effects of the increasing number and size of follicles.

##  Conflict of Interest

There is no conflict of interest that could affect the impartiality of the study conducted.
